# Hypoxia Conditioning for Load-Compromised Athletes: A Narrative Review Exploring Potential Applications in Injury and Disability Management

**DOI:** 10.1007/s40279-025-02322-2

**Published:** 2025-09-30

**Authors:** Wing-Chun Vincent Yeung, Vincent Kwok, Mohammed Ihsan, Olivier Girard

**Affiliations:** 1 Department of Sport Science Research, Taiwan Institute of Sports Science, No. 399, Shiyun Blvd., Zuoying Dist., Kaohsiung City, 813 Taiwan (R.O.C.); 2 Department of Physiotherapy, Institute of Health and Social Care, London South Bank University, London, UK; 3 Physical Education Department, College of Education, United Arab Emirates University, Al Ain, United Arab Emirates; 4 School of Human Sciences (Exercise Sports Science), The University of Western Australia, Perth, WA Australia; 5 Scientific Conditioning Centre, Elite Training Science and Technology Division, Hong Kong Sports Institute, Sha Tin, Hong Kong

## Abstract

**Background:**

Load-compromised athletes are individuals with acute or chronic injuries or disabilities that hinder their ability to perform at peak levels. Hypoxia conditioning is broadly categorized into systemic (i.e., exposure to terrestrial or normobaric hypoxia) or localized (ischemic preconditioning, blood flow restriction training) approaches and could represent a viable option to increase exercise tolerance of load-compromised athletes.

**Purpose:**

This review evaluates the potential of hypoxia conditioning as a training and rehabilitation tool for load-compromised athletes. It explores its applications across various rehabilitation stages and key para-athlete sub-groups including spinal cord injury, limb deficiency, and cerebral palsy.

**Evidence:**

Passive hypoxia conditioning strategies using external limb compression help maintain musculoskeletal function during early rehabilitation stages involving immobilization or minimal loading. As rehabilitation progresses, both systemic and localized hypoxia conditioning (i.e., blood flow restricted exercise) effectively modulates external load while maintaining adequate (internal) physiological strain to induce beneficial cardiometabolic or musculoskeletal adaptations with lower mechanical stress. Para-athletes facing challenges such as biomechanical limitations, reduced active muscle mass, or muscle weakness can benefit from hypoxia conditioning’s capacity to enhance muscle aerobic function, promote muscle strength and hypertrophy, and improve cardiorespiratory performance at lower mechanical loads.

**Conclusion:**

Hypoxia conditioning emerges as a promising intervention to potentially overcome the physical and physiological challenges faced by load-compromised athletes. By addressing their specific limitations, hypoxia conditioning can optimize rehabilitation and training outcomes. Future research is essential to refine hypoxia conditioning protocols and tailor them to maximize individual adaptability and performance across diverse load-compromised athlete populations.

## Key Points

**Table:** 

This review provides an in-depth analysis of systemic and localized hypoxia conditioning as a strategy to address the unique physiological challenges faced by load-compromised athletes, including those rehabilitating from musculoskeletal injuries and para-athletes with spinal cord injuries, cerebral palsy, and limb deficiencies.
The review demonstrates how systemic hypoxia conditioning (e.g., normobaric hypoxia) and localized hypoxia conditioning (e.g., blood flow restriction) enable load-compromised athletes to train at higher relative intensities with reduced mechanical strain, offering a safe and effective method for enhancing recovery, performance, and long-term adaptations.
Hypoxia conditioning modalities deliver significant benefits, including improved muscle oxidative capacity, hypertrophy, strength, and vascular adaptations, effectively countering limitations such as reduced cardiovascular capacity, accelerated muscular fatigue, and diminished muscle strength common in load-compromised athletes.
The paper underscores the need for refined, individualized hypoxia conditioning protocols tailored to specific disability profiles and rehabilitation phases.

## Introduction

Load-compromised individuals represent a diverse group facing challenges in weight-bearing activities due to injuries, medical conditions (e.g., arthritis, obesity), aging, or disabilities [1]. Among them, load-compromised athletes (LCAs) encounter acute or chronic injuries, or disabilities that hinder their ability to train, compete, and perform at their peak.

Musculoskeletal injuries significantly threaten an athlete’s career [2], often requiring extended recovery periods that lead to deconditioning of the cardiovascular, metabolic and muscular systems. For instance, returning to sport following anterior cruciate ligament reconstruction can take 16–52 weeks [3], with losses of up to 5.8 kg in fat-free mass and 0.9–1.5 kg in lean leg mass due to immobilization and inactivity [3, 4]. Even short periods of inactivity (2–8 weeks) can reduce maximal aerobic capacity (*V*O_2max_) by 4–20%, driven by reductions in blood volume, stroke volume, and cardiac output [5]. The financial burden is also considerable, with injuries costing the Swedish National floorball league 315,000 Euros per season [6] and professional English football clubs 125 million Euros annually across 92 clubs (~ 1.4 million Euros per team) [7, 8]. These challenges underscore the urgent need to optimize rehabilitation programs to ensure athletes are adequately prepared to safely return to sport.

Athletes with physical disabilities represent a growing segment of the sporting community, highlighted by the participation of 4400 para-athletes at the Paris 2024 Summer Paralympics [9, 10]. However, research on para-sport remains limited, particularly regarding evidence-based practices for maximizing training outcomes and physiological adaptations within the constraints of physical impairments. Disabilities significantly alter physical performance [11], as seen in para-athletes with spinal cord injuries (SCIs) who experience reduced cardiac dimensions and muscle mass engagement, leading to diminished performance outcomes [12]. Likewise, [13] conditions such as multiple sclerosis contribute to muscle weakness and decreased mobility [14, 15]. These barriers highlight the pressing need for safe and effective strategies to support optimal training adaptations in this population.

**Fig. 1:**
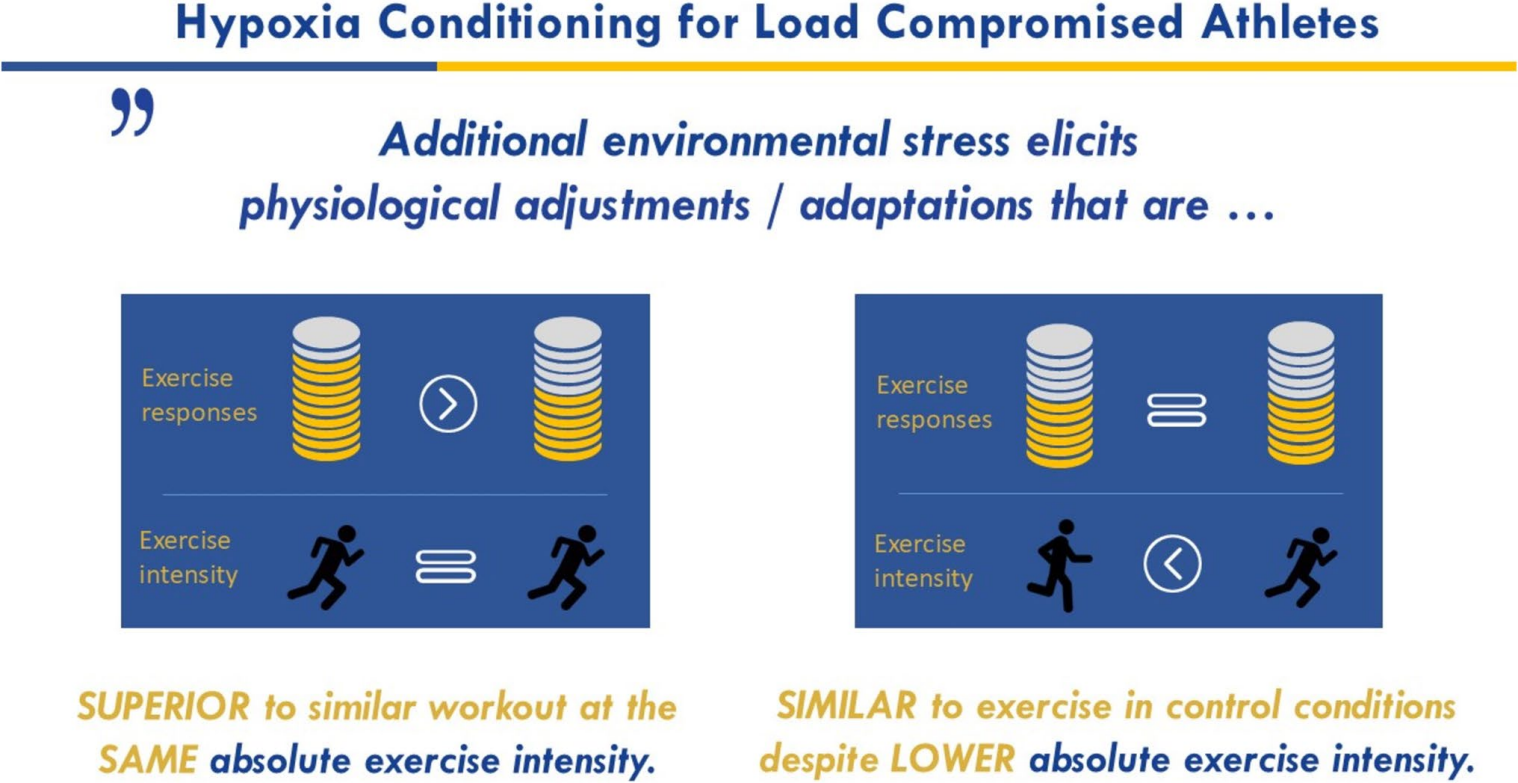
Physiological rationale for prescribing hypoxia conditioning among load-compromised athletes

Hypoxia conditioning (HC) offers a promising solution to increase cardiometabolic intensity without significantly raising mechanical strain on the locomotor system. This allows individuals to exercise at lower intensities, reducing mechanical strain (i.e., external load) while achieving comparable physiological responses (i.e., internal load) to normoxic training (Fig. 1). HC involves exposure to systemic (breathing low oxygen mixtures) and/or localized (external limb compression such as ischemic preconditioning [IPC] or blood flow restriction [BFR]) hypoxia, either passively or in conjunction with exercise [16]. Traditionally used to improve endurance performance through altitude training [17], HC is now gaining recognition as a rehabilitation intervention, benefiting body weight management, and enhancing glucose and lipid metabolism, even at low exercise intensities [18]. By adding hypoxic stress, HC can provide a stronger training stimulus (i.e., cardio-metabolic solicitation), potentially preserving cardiorespiratory fitness when regular training is restricted (Fig. 1).

This narrative review examines HC as a therapeutic intervention for rehabilitating and para-athletes, collectively termed LCAs. It briefly outlines the physiological adaptations elicited by both systemic and localized HC, highlighting their relevance to aerobic and strength development. This review examines the emerging yet limited literature on HC modalities, focusing on their impact on rehabilitation and performance outcomes in LCAs, while also identifying key research gaps. Finally, it provides evidence-based insights to optimize HC applications for training and recovery in this unique population.

## Physiological Adaptations Following HC

### Systemic HC

Systemic HC induces both hematological and non-hematological adaptations. Hematological adaptations include red blood cell expansion, which enhances the blood’s O_2_-carrying capacity. Reduced O_2_ partial pressure in hypoxic environments increases hypoxia inducible factor-1 expression [19], which regulates erythropoietin gene transcription [20], stimulating red blood cell production. These adaptations enhance oxygen delivery, leading to improvements in aerobic capacity. Hypoxia inducible factor-1 activation also triggers several non-hematological adaptations by regulating multiple genes such as vascular endothelial growth factor, monocarboxylate transporters 1 and 4, nitric oxide synthase, and carbonic anhydrase [21]. These adaptations enhance capillary function, vascular health, glucose regulation, lactate metabolism, and pH buffering capacity [21].

While systemic HC is traditionally used for aerobic adaptations, recent interest has focused on its potential to enhance hypertrophy and muscle strength [22]. Proposed mechanisms include hypoxia-induced increases in anabolic factors such as growth hormone [23] and insulin-like growth factor-1 [24, 25], as well as stimulation of early myogenesis through elevated myogenin factor 5 and myoblast determination protein 1 messenger RNA content [26]. However, research outcomes remain inconsistent. While some studies [23–26] have reported increased muscle strength or hypertrophy (e.g., cross-sectional area [CSA], muscle thickness) when resistance training is combined with HC, others [27, 28] have found no significant benefits. A recent meta-analysis reported comparable improvements in muscle CSA and one-repetition maximum (1RM) performance between resistance training combined with HC and normoxia [29]. However, sub-analyses indicated that HC with moderate loads (60–80% 1RM) and shorter (≤ 60 s) inter-set rest intervals was more effective for promoting hypertrophy, while longer inter-set rest intervals (≥ 120 s) better supported strength gains [29]. Overall, systemic HC may confer hypertrophic and strength benefits over normoxic training, although individual responses and optimal hypoxic dosing play a key role.

### Localized HC

Localized HC modalities, including IPC and BFR training, have shown promising effects. Ischemic preconditioning is recognized for its acute performance enhancements [30] and potential to promote angiogenic adaptations following repeated applications [31]. For instance, in healthy humans, a 7-day bilateral lower-limb IPC protocol (four cycles of 5-min occlusion at 220 mmHg and 5-min reperfusion) improved skeletal muscle oxidative capacity, microvascular blood flow kinetics, and recovery from submaximal exercise [32], as well as enhanced submaximal cycling efficiency and endurance performance [33]. These findings underscore the potential of IPC as a passive HC strategy to improve muscle aerobic function in both clinical and athletic performance contexts.

Blood flow restriction training uniquely combines endurance and resistance benefits, eliciting both hypertrophic and oxidative adaptations [34]. This dual effect makes localized HC an attractive option for LCAs by maintaining or minimizing physical fitness declines during periods of reduced training loads because of injury recovery or augmenting training stimuli constrained by disabilities. For example, in healthy men, Conceição et al. [34] demonstrated that combining BFR with low-intensity cycling (40% *V*O_2max_) significantly improves muscle CSA, 1RM leg press strength, and ˙*V*O_2max_, with gains comparable to traditional leg-press resistance exercise and endurance training at 70% *V*O_2max_. Possible mechanisms include activation of anabolic pathways like the Akt-mTOR cascade [35, 36], increased ribosomal biogenesis leading to improved translational capacity [35], and satellite cell activation [37]. Blood flow restriction training also induces oxidative adaptations including increased oxidative capacity [38], likely mediated by transcriptional coactivator PGC-1α [39] and enhanced vascular adaptations via vascular endothelial growth factor [40], a downstream target of both PGC-1α and hypoxia inducible factor-1 [41–43].

## Physiological Rationale for Prescribing HC Among LCAs

Advances in HC research demonstrate that positive physiological adaptations can occur with minimal or no contractile activity, making it particularly advantageous for LCAs with movement limitations. Systemic HC increases submaximal *V*O_2_ for a given absolute workload, eliciting greater physiological and perceptual responses [44]. Likewise, exercising at a set intensity (e.g., 75% *V*O₂_max_) under hypoxia reduces external power output [45], reflecting the well-documented decline in aerobic capacity. In healthy fit individuals, and in the absence of prior acclimation, *V*O_2max_ on average decreases by ~ 6.3% per 1000 m of altitude ascent due to decreased partial pressure of inspired O_2_ [46].

Exercising with BFR, similar to systemic hypoxia, increases physiological strain while maintaining low mechanical output, comparable to unrestricted conditions. By setting a predetermined tolerable mechanical load (e.g., walking speed or cycling power output), varying cuff pressure modulates internal and external loads [47]. Research demonstrates that applying BFR at 60% of arterial occlusion pressure (AOP) during walking at speeds of 6–7.2 km·h⁻^1^ elevates cardiorespiratory responses to levels comparable to unrestricted equivalents [48]. Continuous BFR during walking can exacerbate fatigue and alter gait kinematics, requiring careful consideration when designing exercises, especially for individuals with compromised gait or those recovering from lower limb injuries [49]. Alternatively, combining heart rate (HR) clamping with different %AOPs can effectively manage external workloads. For instance, clamping HR at the first ventilatory threshold and applying cuff pressures above 45% AOP significantly reduces external power output by 15–30% compared with unrestricted conditions, while increasing muscle deoxygenation and blood pooling—effects that are exaggerated at 75% AOP [50].

Ensuring HC is well tolerated by LCAs, who may already experience discomfort from injuries or disabilities, is crucial for fostering compliance and successful integration into training periodization. Effectively managing the perceptual demands of HC while balancing its physiological benefits can optimize performance and rehabilitation outcomes for LCAs.

## HC and the Rehabilitating Athlete

Rehabilitation is key for recovering from athletic injuries and restoring pre-injury performance levels. While early rehabilitation focuses on pain relief, swelling reduction, and joint mobility, addressing muscle atrophy and strength deficits is equally crucial to prevent long-term dysfunction [51, 52] including knee osteoarthritis [53, 54] and the reduced likelihood of returning to peak performance [55, 56]. Minimizing strength deficits early in rehabilitation not only facilitates recovery but also reduces reinjury risks [57]. Consequently, effective management of both cardiovascular and muscle functions is essential for a successful return to sport and minimizing reinjury [58]. Inadequate rehabilitation or a premature return to sport, particularly without an objective evaluation, can prolong recovery and increase the chance of subsequent injury [56, 59]. Hypoxia conditioning presents a valuable rehabilitation tool for LCAs to minimize mechanical stress on the musculoskeletal system while providing the necessary physiological stimulus. This approach supports an efficient and timely return to sport, a crucial outcome in high-performance sport.

### Localized HC

**Fig. 2:**
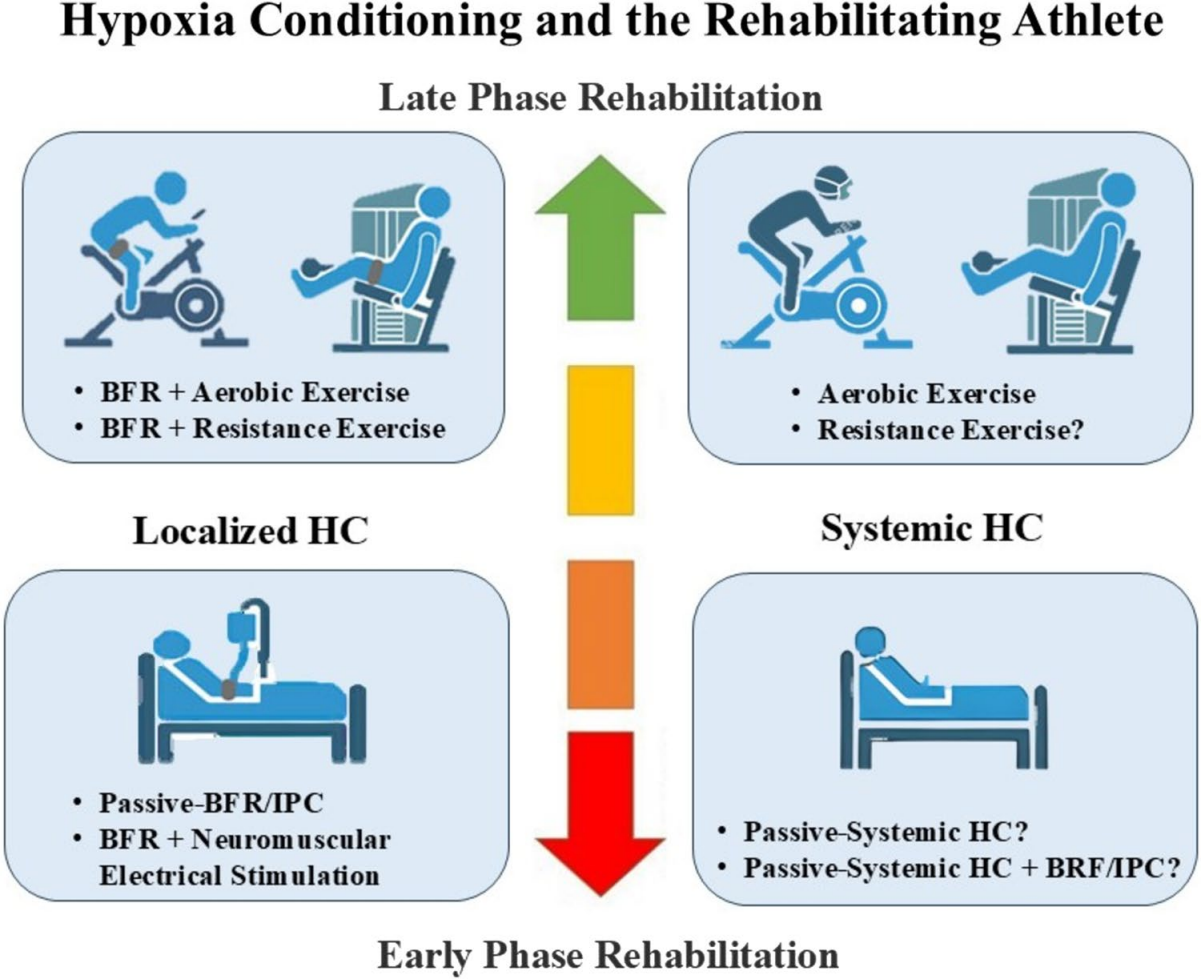
Conceptual use of localized and systemic hypoxia conditioning (HC) modalities during rehabilitation. *BFR* blood low restriction, *IPC* ischemic preconditioning

Localized modalities such as IPC or BFR training have shown significant potential in improving musculoskeletal function, especially during early post-surgery recovery (Fig. 2). Blood flow restriction potentially supports early-stage rehabilitation efforts by reducing the need for progressive mechanical loading, thereby minimizing muscle atrophy and strength loss. Continuous BFR (200–240 mmHg) over 10–14 days has been shown to reduce leg muscle atrophy and strength loss in patients recovering from ACL reconstruction or those undergoing cast immobilization for 14 days [60, 61]. Additionally, Kubota et al. [62] demonstrated that occlusion pressures as low as 50 mmHg could prevent muscle strength and mass declines. In their study, five sets of 5-min BFR cycles (interspersed with a 3-min recovery) performed twice daily for 2 weeks of cast immobilization prevented declines in knee extensor-flexor torque. Likewise, repeated bilateral lower-limb IPC (four cycles of 5-min occlusion [220 mmHg], 5-min reperfusion) across 7 consecutive days demonstrated improved skeletal muscle oxidative capacity and microvascular blood flow kinetics, facilitating rapid recovery from submaximal exercise [32]. While both studies were conducted in healthy individuals, these findings support the use of passive BFR and IPC as early-stage rehabilitation strategies, potentially benefiting LCAs during periods of immobilization or reduced loading.

In cases of prolonged immobilization due to injury complications or multiple surgical procedures, combining neuromuscular electrical stimulation (NMES) with BFR may offer benefits. Although research on athletes is limited, studies among healthy individuals indicate that NMES and BFR over multiple sessions can enhance quadriceps and soleus muscle size, strength, and CSA [63–66]. Although the optimal frequency of NMES-BFR application remains unclear, studies commonly delivered more than 20 sessions over 2 weeks. Pending confirmatory research, this approach could be particularly effective during early rehabilitation phases when joint loading is restricted.

As athletes progress in recovery and begin to load their musculoskeletal system, BFR combined with external loading is preferred over NMES and BFR. Research indicates that low-load resistance training with BFR (LL-BFR) enhances vastus lateralis CSA more effectively than NMES and BFR, making it suitable for LCAs once loading is well tolerated [67]. In young adults recovering from ACL reconstruction, LL-BFR with external loads up to 30% 1 RM improves quadriceps muscle strength and muscle mass compared with non-BFR training [68]. Moreover, LL-BFR may elicit vascular and mitochondrial adaptations, helping to preserve muscle aerobic function as LCAs transition back to full fitness [69]^]^.

Another advantage of LL-BFR is its ability to modulate pain, making it beneficial for LCAs in rehabilitation. Compared with unrestricted controls, integrating BFR into physiotherapy sessions alleviates knee pain immediately and up to 24 h post-session in patients with an ACL reconstruction [70]. Likewise, LL-BFR has been shown to reduce acute anterior knee pain during a single physiotherapy session [71, 72] and alleviate chronic pain during daily activities for up to 8 weeks among patients with patellofemoral pain [73]. By reducing pain and facilitating progressive loading, LL-BFR provides a structured pathway for LCAs to progress through rehabilitation without placing excessive mechanical strain on injured joints.

For LCAs progressing to light aerobic training and weight-bearing activities, incorporating BFR training into low-intensity aerobic exercise may help preserve cardiorespiratory fitness while minimizing strain on injured areas (Fig. 2). Although research on injured athletes is limited, studies in healthy individuals suggest that low-intensity aerobic training with BFR enhances *V*O_2max_ compared with training without BFR. For instance, collegiate basketball players demonstrated an 11% *V*O_2max_ increase after 24 sessions of treadmill walking (4–6 km·h^−1^ at 5% incline, 5 sets of 3 min) with BFR over 2 weeks [38]. Similarly, trained swimmers and elite rowers improved *V*O_2max_ by ~ 9% when BFR was added to low-intensity training sessions (i.e., lactate threshold 1) totaling 300 min over 5 weeks [74, 75]. The low-load nature of BFR-enhanced aerobic training is well suited for athletes in latter rehabilitation stages, reducing strain on healing tissues while providing an effective cardiovascular stimulus. Tailored BFR protocols aligned with injury status and rehabilitation goals of each LCA can optimize recovery by safely supporting both strength and aerobic improvements.

### Systemic HC

Training in normobaric hypoxia is a well-established systemic HC model used to improve cardiovascular fitness, particularly among endurance athletes aiming to boost red blood cell mass and O_2_-carrying capacity for superior sea-level performance [17]. Systemic HC now encompasses several methods, from passive exposure to submaximal and supramaximal exercise at varying hypoxia levels, as well as continuous or intermittent exposure during exercise or recovery phases only, to optimize physiological adaptations [76, 77], or promote soft-tissue healing following injury [78]. These approaches are not exclusive to elite athletes and have also been applied to clinical populations and individuals with weight-bearing limitations. For example, systemic HC has been utilized in geriatric patients with multiple comorbidities to maintain relative exercise intensities (i.e., 80% maximal HR) while decreasing the absolute workload by 25% [79]. Passive exposures (i.e., 60-min continuous or 5–9 cycles of 3–5 min hypoxic-normoxic intervals) at fraction of inspired oxygen ~ 12–15% over 3–4 weeks (five sessions per week) have improved exercise performance in healthy sedentary men [80] and patients with mild chronic obstructive pulmonary disease [81]. Additionally, low-load (i.e., 20% 1RM) resistance training in hypoxia (blood oxygen level ~ 80%) over 5 weeks (three-weekly sessions) yielded greater muscle strength and endurance gains than normoxic training among netball players [82, 83]. While these studies do not directly confirm the efficacy of systemic HC for LCAs, they offer proof-of-concept for its potential in facilitating the transition back to load-bearing exercises. Case reports, such as one involving a professional female handball player undergoing an ACL pre-operative rehabilitation, suggest systemic HC may facilitate recovery [84]. The rehabilitation program included several micro-cycles of unilateral exercise and running in normobaric hypoxia (15% fraction of inspired oxygen), totaling approximately 100 min of exposure. The hypoxia-induced elevation of growth hormone and erythropoietin levels likely supported the athlete’s return to a full training load and competitive sport [84]. However, randomized clinical trials are necessary to confirm the clinical efficacy of systemic HC and establish optimal protocols for its integration into pre- or post-operative rehabilitation.

## HC and the Para-Athlete

While HC benefits are well documented for able-bodied athletes, its application in para-athletes remains under-researched. Implementing HC for this LCA cohort requires addressing unique challenges, such as altered biomechanics, potential compensatory injuries, and specific contraindications. Individualized HC protocols are crucial, as the International Paralympic Committee recognizes ten distinct impairment types that determine para-athlete eligibility [85]. This section reviews the limited literature on HC in para-athletes, focusing on both systemic and localized HC approaches. Emphasis is placed on major para-athlete classifications, SCI, limb deficiency, and cerebral palsy (CP), as these represent key sub-groups within the para-athlete community [86].

## Para-Athletes with SCI: Physiological Challenges and the Potential for HC

Para-athletes with SCI face unique challenges because of altered cardiorespiratory, metabolic, neuromuscular, and thermoregulatory functions, leading to reduced physiological and functional capacities. The severity and location of the injury significantly influence these limitations. Athletes with tetraplegia (C5–C8) and high paraplegia (T1–T6) generally demonstrate reduced cardiovascular and sudomotor functions compared with able-bodied athletes [86]. In contrast, athletes with lower paraplegia often exhibit decreases in tidal volume, minute ventilation, O_2_ uptake, and stroke volume, typically accompanied by increased HR. Additionally, athletes with SCI have reduced active muscle mass available for exercise. Most rely on upper body musculature for wheelchair sports, demanding a higher percentage of power output relative to muscle mass. This results in accelerated fatigue from intracellular metabolite accumulation (e.g., H+, lactate, and inorganic phosphate) [87, 88]. Furthermore, upper body muscles contain a higher proportion of less oxidative fibers, making them less efficient at O_2_ extraction and circulation [89], and exhibit lower fat oxidation compared to leg musculature, thereby increasing reliance on anaerobic metabolism [90].

**Fig. 3:**
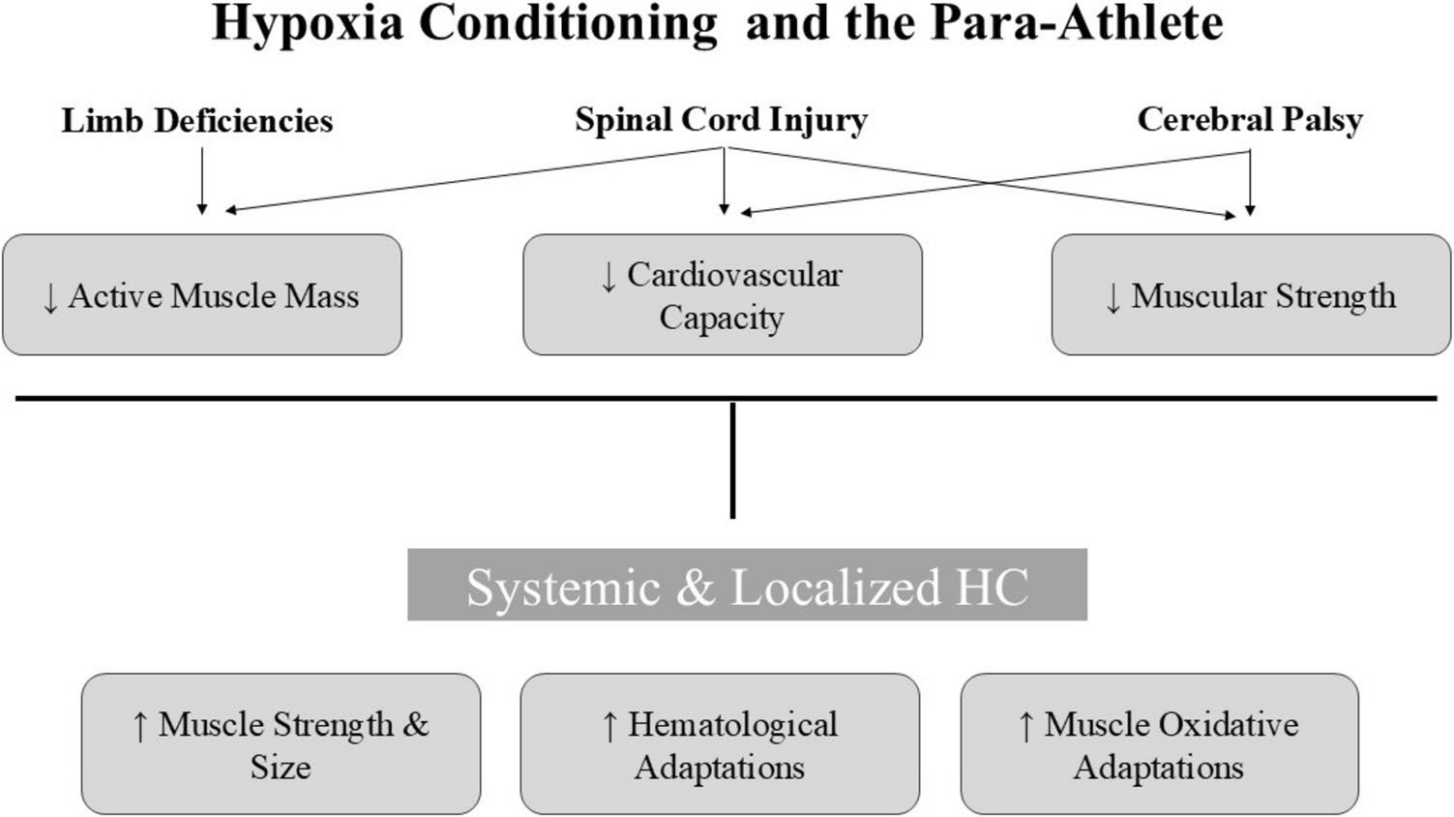
Conceptual framework illustrating the potential of systemic and localized hypoxia conditioning (HC) modalities to address the physiological challenges faced by para-athletes with limb deficiencies, spinal cord injuries, and cerebral palsy. Key limitations, including reduced exercising muscle mass, diminished cardiovascular capacity, and decreased muscular strength (*arrows* indicate specific impairments), can be mitigated through HC. Systemic and localized HC modalities collectively drive physiological adaptations, such as enhanced muscle hypertrophy and strength, improved muscle oxidative capacity, and augmented central hematological adaptations, offering targeted strategies to overcome functional limitations, ↑ and ↓ for increased and decreased, respectively

The potential for HC to mitigate compromised cardiorespiratory and musculoskeletal responses in athletes with SCI is gaining recognition, despite limited research in this area. Both systemic and localized HC modalities are known to increase cardiovascular intensity and stimulate muscular adaptations, even with reduced active skeletal muscle mass. Systemic HC approaches, such as training in normobaric hypoxia, may allow athletes with SCI to train at greater relative intensities, thereby partially offsetting the inherent limitations of reduced muscle mass during upper-body exercises. Additionally, localized HC such as BFR training may provide additional benefits by triggering anabolic processes, including the release of human growth hormone [91] and downregulation of myostatin expression [92]—both key mediators of muscle hypertrophy and strength development. Emerging evidence also suggests that BFR training can augment vascular and mitochondrial adaptations [32, 69]. These mechanisms collectively indicate that HC can augment muscle mass, strength, and aerobic function in both paralyzed and active muscles, offering a promising approach for enhancing performance in para-athletes with SCI (Fig. 3).

### Systemic HC

Research on systemic HC in para-athletes remains limited, but existing findings highlight its potential to enhance training outcomes. However, studies involving para-cyclists with various impairments, including SCI, have implemented altitude exposures via normobaric hypoxia (2000–4000 m) over 2-week periods without reporting adverse effects [93, 94]. These performance gains occurred without changes in O_2_ transport capacity, *V*O_2max_, or ventilation, but with a reduction in the blood lactate concentration [93, 94]. Researchers attributed these improvements to non-hematological mechanisms, such as enhanced mitochondrial and capillary adaptations, while acknowledging that 2 weeks may be insufficient to elicit changes in maximal exercise capacity. While these studies demonstrate that systemic HC may improve endurance performance in para-athletes with SCI, their findings are limited by small sample sizes (*n* = 3–6) and the lack of sea-level control comparisons. Consequently, high-quality trials involving elite para-athletes are still needed to confirm the efficacy of systemic HC in this LCA cohort. In the meantime, practitioners should closely monitor physiological responses and performance under both normoxic and hypoxic conditions to tailor exposure and assess individual adaptations.

Para-athletes with SCI often exhibit reduced respiratory function [86], which may limit their ability to cope with increased ventilatory demands associated with hypoxia. This limitation could be exacerbated when systemic HC is combined with training-induced increases in body temperatures [95]. High or unaccustomed HC stimuli may increase the risk of hypoventilation, hypoxemia, and premature fatigue [96]. However, studies involving para-cyclists with various impairments, including SCI, have implemented simulated altitude exposures (2000–4000 m) over 2-week periods without reporting adverse effects [93, 94]. These findings suggest that, when applied carefully, systemic HC is feasible in this cohort. Ultimately, practitioners must adopt a cautious, individualized, and progressive approach to determine suitable HC dosing for athletes with SCI and other load-compromised conditions.

### Localized HC

For para-athletes with SCI engaging in wheelchair sports, upper limb muscle strength and endurance are crucial for performance [11]. Blood flow restriction has shown promise in simultaneously promoting hypertrophic and oxidative adaptations [34], rendering it an attractive conditioning strategy for para-athletes who rely heavily on upper body strength. Although research specifically examining BFR benefits in SCI populations is limited, particularly among LCAs with SCI, existing studies offer promising findings.

Incorporating BFR during exercise can enhance muscle hypertrophy in both affected and unaffected limbs. In healthy men, Conceição et al. [34] demonstrated that adding BFR to low-intensity cycling (40% *V*O_2max_) improved both cardiovascular (i.e., *V*O_2max_) and strength indices (1RM leg press), highlighting its potential to elicit adaptations across both aerobic and strength domains. Similarly, studies on individuals with SCI indicate that both low-intensity (30% 1RM) exercise or electrical stimulation of the forearms (20 Hz, 450-μs pulse duration, 5 s on/5 s off contraction-relaxation) combined with BFR (30–60% of forearm occlusion pressure) increased forearm CSA and muscle strength over 6–8 weeks of twice-weekly sessions [97, 98]. However, despite these increases in CSA, the adaptations did not translate to improved functional performance during grasp-and-release tasks [97]. Moreover, Skiba et al. [99] demonstrated that functional electrical stimulation (20 Hz, 400-μs pulse duration, 15 s on/4 s off cycle) combined with BFR (average 65.2 ± 7 mmHg) enhanced rectus femoris and vastus medialis muscle thickness after 16 training sessions over 8 weeks in individuals with complete SCI.

An important consideration when using BFR in individuals with SCI is the increased risk of venous thromboembolism, owing to the formation of small collateral veins that may obstruct major deep veins in the legs [100]. Consequently, individuals should be screened for venous thromboembolism before undergoing BFR, and prophylactic measures should be considered to mitigate this risk [100]. In a study conducted over 3 years on patients with SCI cleared of venous thromboembolism, BFR interventions did not result in any cases of acute autonomic dysreflexia, deep vein thrombosis, pain, or reductions in total work output during knee extensor exercises [101]. As such, preliminary findings indicate that, when performed under appropriate medical supervision, BFR may be a promising strategy to improve muscle hypertrophy and endurance in upper body muscles of para-athletes with SCI.

## Para-Athletes with Limb Deficiency: Physiological Challenges and Potential Interventions

Para-athletes with limb deficiencies represent a significant segment of Paralympic competitors, with classifications based on the extent and location of missing limb segments [102]. Most athletes in this category utilize prosthetics, wheelchairs, or other adaptive equipment, although prosthetics are prohibited in certain sports such as swimming. Unlike other para-athlete groups, individuals with limb deficiencies do not face cardiorespiratory or autonomic limitations, nor do they experience central nervous system impairments associated with their disability [86, 103]. The primary physiological challenge for this cohort is the reduced muscle mass available for exercise, which increases the load on the remaining limbs. This often leads to elevated blood lactate levels [104], higher energy demands during exercise [105], and decreased movement efficiency [105], with the severity of these issues influenced by the type of surgical procedure and level of amputation [105].

### Systemic HC

Although para-athletes with limb deficiencies generally demonstrate physiological responses comparable to able-bodied athletes, research on systemic HC and its effects on their physiological capacity is limited. Given their unaffected cardiorespiratory and autonomic responses, they may benefit from systemic HC similarly to other athlete populations. In support, studies on para-cyclists with various disabilities, including limb deficiencies, demonstrated improved cycling time-trial performance (3–14 km) following 2 weeks of training at simulated altitudes between 2000 and 4000 m [93, 94]. Further, a case study of an elite wheelchair marathoner with Charcot-Marie-Tooth disease reported improved 3-km time trial performance (power: + 3–6 W, time: − 27–216 s) following an 8-week terrestrial altitude training program at 3860 m [106]. Although evidence is limited, systemic HC may enhance endurance performance and physiological adaptations in para-athletes with limb deficiencies. However, the absence of adequate control groups in a small number of clinical trials available limits conclusions about its true efficacy.

### Localized HC

Para-athletes with limb deficiencies face challenges associated with reduced muscle mass, leading to greater reliance on the remaining active muscles, greater fatigue, increased energy expenditure, and greater strain during exercise. A case study by Prescott et al. [107] involving a 36-year-old transtibial amputee demonstrated significant improvements in muscle strength, function, and performance, including after 12 weeks of BFR training across 28 sessions. Enhanced outcomes were observed in the *L*-Test and 2-min walk test, highlighting BFR training’s potential as an effective localized hypoxic stimulus for muscle adaptation in para-athletes with prosthetics. While research on BFR in para-athletes remains limited, evidence suggests it is a promising modality, particularly for wheelchair-dependent athletes or those heavily reliant on specific muscle groups. Properly implemented localized HC methods such as BFR potentially improve strength, endurance, and functional capacity, helping para-athletes overcome challenges imposed by reduced muscle mass.

## Para-Athletes with CP: Physiological Challenges and Potential Interventions

Cerebral palsy is characterized by movement impairments and diminished muscle strength resulting from static brain lesions during development. These neurological impairments arise from pyramidal and extrapyramidal lesions, each contributing to distinct motor deficits [108]. Pyramidal lesions result in spasticity, hypertonia, and exaggerated deep tendon reflexes, which impede voluntary muscle control and increase muscle stiffness. Extrapyramidal lesions, however, affect muscle tone regulation, coordination, and postural control [19]. Individuals with CP typically experience reduced muscle strength, aerobic capacity, and agility [109]. Research on non-athletes with CP indicates their aerobic capacity is 21–61% lower than non-CP peers [110, 111]. Among children with CP, peak power during a 20-s Wingate test is 27–46% lower and repeat sprint agility task completion times are 39% longer compared with non-CP children [112]. Similar trends are seen among elite CP athletes, such as footballers and cyclists, who achieve similar *V*O_2peak_ and corresponding peak power during incremental cycling tests but exhibit 31–47% lower knee extension strength and 33% reduced mechanical output during a 30-s Wingate test [113]. These observations highlight the importance of muscle strength as a key determinant of athletic performance in para-athletes with CP. Despite aerobic performance approaching that of non-CP athletes, significant deficits in leg muscle strength and power remain. Strong associations between muscle strength and both sprint and peak aerobic power underscore the necessity of targeted interventions to enhance strength in athletic performance in this population [113].

### Systemic HC

Aerobic capacity is highly trainable in individuals with CP through modalities including treadmill walking/running, cycling, or arm ergometry [109]. Although strength impairments [113] may contribute to movement limitations and abnormal gait patterns, the overall physiological responses in individuals with CP are generally comparable to able-bodied individuals owing to the absence of major neuroanatomical disruptions. Therefore, both hematological and non-hematological adaptations associated with systemic HC in able-bodied athletes are likely applicable to para-athletes with CP (Fig. 3). However, reduced functional lung volumes and respiratory muscle weakness are common in individuals with CP [114]. This limitation may compromise their ability to meet increased ventilatory demands associated with hypoxia, notably when coupled with elevated body temperatures during training [95]. Exposure to a high or unaccustomed HC stimulus may increase the risk of hypoventilation, hypoxemia, and premature fatigue [96]. When prescribing exercise for individuals with CP, careful monitoring of pathological fatigue, muscle spasms, and maximal exertion is crucial, along with a thorough assessment of physical capacity [108]. Because of the variability in HC effects related to individual differences and brain injury severity, a progressive approach is essential to minimize risks and adverse outcomes when implementing HC as a training intervention.

Current evidence regarding the use of systemic HC in para-athletes with CP is limited, primarily derived from studies with small sample sizes (i.e., *n* = 2) conducted within broader investigations involving para-cyclists with various disabilities [93], 94. Notably, 2 weeks of training at simulated altitudes of 2000–4000 m enhanced *V*O_2max_ (+ 1.2 ml·kg^−1^·min^−1^) and corresponding power output (+ 25 W), leading to a 67-s improvement in a 14-km cycling time trial. Similarly, a study involving six national para-cyclists with diverse disabilities (including one cognitively impaired athlete) found that 2 weeks of systemic HC at simulated altitudes of 2000–3000 m improved 3-km cycling time-trial performance and reduced post time-trial blood lactate levels, despite no changes in *V*O_2max_ [93].

These findings indicate that systemic HC may enhance performance in para-athletes with CP. However, the absence of sea-level controls in these studies impairs attributing these improvements solely to HC-specific adaptations. Further, it is essential to consider whether a systemic HC model effectively addresses the primary limiting factors within this cohort. Muscle strength has been identified as a primary limiting factor for individuals with CP [113], yet evidence on strength gains from systemic HC interventions remains inconsistent [22]. Given that aerobic capacity is a trainable attribute in this population [109], combining targeted interventions for strength and aerobic training may be necessary to optimize performance outcomes for para-athletes with CP.

### Localized HC

Localized HC, particularly BFR training, appears well suited for addressing key performance-limiting factors in LCAs with CP (Fig. 3). A substantial body of literature supports the efficacy of BFR in promoting strength gains when combined with low-load resistance training [115]. This approach seems particularly relevant for athletes with CP, who often present with strength deficits and are limited in their ability to perform high-load resistance exercises [109, 113]. Emerging evidence underscores the potential of BFR in this context, where an 8-week BFR (60–80% AOP) training regimen combined with strength training improved three-repetition maximum leg press and extension strength, as well as the functional sit-to-stand test in three male individuals with CP, despite no changes in isometric muscle force and muscle mass [116]. Similar findings in children with CP show that BFR combined with conventional strength, balance, and core exercises increased muscle thickness in both the trunk (external/internal obliques, and gluteus maximus) [117] and lower limbs (rectus femoris, gastrocnemius, and gluteus medius) [118]. These findings suggest that BFR may be a viable training strategy for LCAs with CP. By addressing barriers to high-load resistance training, BFR training enables athletes with CP to reap the associated training adaptations [119].

Despite its therapeutic potential, BFR carries risks such as physical discomfort and transient blood pressure elevations. However, recent work in individuals with CP shows that blood pressure remained within safe clinical thresholds (i.e., systolic < 200, diastolic < 110 mmHg) during low-load resistance training (15–25% 1RM) combined with BFR at 60–80% AOP [116]. While participants initially reported occlusion-related discomfort, particularly during the first week, this sensation diminished over time, with most adapting by week 4 and reporting minimal discomfort throughout the remaining 8-week intervention [116]. To reduce early intolerance, a gradual introduction of BFR is recommended in CP populations, beginning with lower occlusion pressures and fewer repetitions to facilitate neuromuscular and perceptual adaptations [116].

## Limitations

The authors acknowledge that much of the evidence supporting HC use stems from studies involving healthy able-bodied individuals under controlled experimental conditions. In contrast, evidence from LCAs relevant to this review is considerably more limited. Available studies are often case reports [84, 106, 107], field-based investigations [93, 94], or small pilot trials that frequently lack adequate control groups [93, 94, 101, 116], blinding procedures that could influence performance outcomes, or adequate sample sizes [93, 94, 116]. While we recognize the inherent challenges of conducting rigorously controlled trials within LCA populations, these limitations underscore the need for well-designed, adequately powered clinical studies to determine the efficacy and safety of HC applications in this unique cohort. Additionally, while HC interventions may help mitigate cardiovascular deconditioning and muscle atrophy, or facilitate muscle mass maintenance during periods of injury or reduced loading, evidence remains limited regarding their impact on key performance determinants such as contraction velocity or neuromuscular coordination. These factors are essential for power output in most sports, warranting further research to elucidate the functional benefits of HC beyond muscle mass preservation.

## Conclusions

Therapeutic use of hypoxia through HC offers innovative solutions for managing LCAs, including athletes and para-athletes, by addressing physical and physiological limitations. Its core principle is modifying the relationship between external and internal loads (Fig. 1). Traditionally implemented to enhance physiological and perceptual responses through systemic or localized hypoxia conditioning, HC now emphasizes reducing the total workload during exercise during a fixed internal load exercises (e.g., clamped HR or perceptually regulated tasks), achieving comparable (or even greater) cardiometabolic benefits while minimizing mechanical stress.

Both systemic and localized HC offer solutions to challenges such as deconditioning, muscle atrophy, and cardiovascular decline during rehabilitation, immobilization, or adaptive training. Systemic HC promotes hematological and non-hematological adaptations, reducing mechanical strain and maintaining cardiovascular fitness during inactivity. Localized HC, particularly with BFR, boosts muscle strength and hypertrophy, countering muscle atrophy in LCAs. These protocols likely reduce perceptual strain and enhance compliance, enabling their safe integration into structured rehabilitation and training programs. Tailored HC interventions could minimize reinjury risks, accelerate recovery, and support a gradual return to regular exercise. These approaches can also address the specific needs of para-athletes, such as those with SCI or CP, enriching their strength and conditioning toolbox (Fig. 3).
